# Supplementary material for: In Vitro and In Silico Antimalarial Evaluation of FM-AZ, a New Artemisinin Derivative
Source: Medicines (Basel). 2022 Jan 24;9(2):8. doi: 10.3390/medicines9020008 (PMC8880451; doi:10.3390/medicines9020008)
Supplement: Supplementary file 1 [file medicines-09-00008-s001.zip › medicines-1514831-supplementary.pdf]

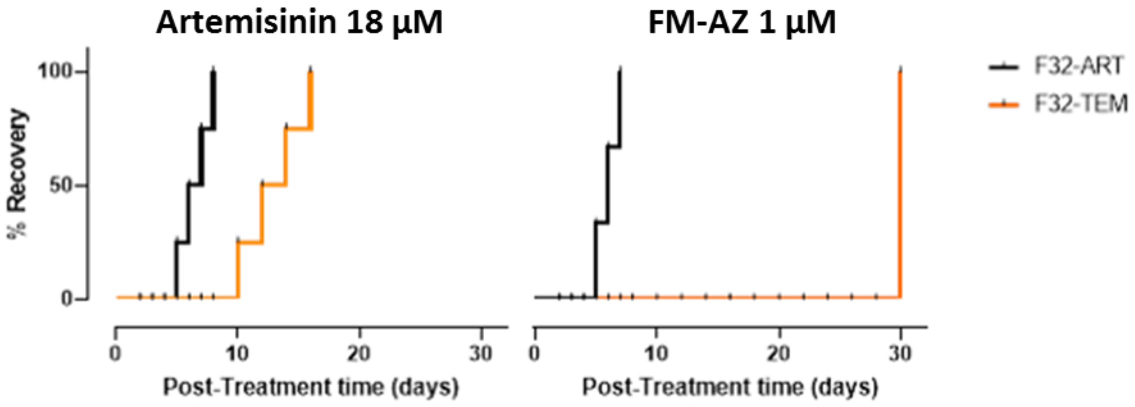

Figure S1: Efficacy of artemisinin and FM-AZ with regards to artemisinin-sensitive and artemisinin-resistant Plasmodium falciparum via recrudescence curves
